# Circulating fatty acid-binding protein 1 (FABP1) and nonalcoholic fatty liver disease in patients with type 2 diabetes mellitus

**DOI:** 10.7150/ijms.40417

**Published:** 2020-01-14

**Authors:** Yung-Chuan Lu, Chi-Chang Chang, Chao-Ping Wang, Wei-Chin Hung, I-Ting Tsai, Wei-Hua Tang, Cheng-Ching Wu, Ching-Ting Wei, Fu-Mei Chung, Yau-Jiunn Lee, Chia-Chang Hsu

**Affiliations:** 1 Division of Endocrinology and Metabolism, E-Da Hospital, Kaohsiung, 82445 Taiwan; 2 Division of Cardiology, E-Da Hospital, Kaohsiung, 82445 Taiwan; 3 Division of Gastroenterology and Hepatology, Department of Internal Medicine, E-Da Hospital, Kaohsiung, 82445 Taiwan; 4 Department of Obstetrics & Gynecology, E-Da Hospital, Kaohsiung, 82445 Taiwan; 5 Departmen of Emergency, E-Da Hospital, Kaohsiung, 82445 Taiwan; 6 Division of General Surgery, Department of Surgery, E-Da Hospital, Kaohsiung, 82445 Taiwan; 7 Lee's Endocrinology Clinic, Pingtung, 90000 Taiwan; 8 School of Medicine, College of Medicine, I-Shou University, Kaohsiung, 82445 Taiwan; 9 The School of Chinese Medicine for Post Baccalaureate, College of Medicine, I-Shou University, Kaohsiung, 82445 Taiwan; 10 Division of Cardiology, Department of Internal Medicine, E-Da Cancer Hospital, Kaohsiung 82445 Taiwan; 11 Department of Obstetrics & Gynecology, E-Da Dachang Hospital, Kaohsiung 80794 Taiwan; 12 Health Examination Center, E-Da Dachang Hospital, Kaohsiung, Taiwan

**Keywords:** Fatty acid-binding protein 1, nonalcoholic fatty liver disease, type 2 diabetes mellitus

## Abstract

**Background**: Fatty acid-binding protein 1 (FABP1) (also known as liver-type fatty acid-binding protein or LFABP) is a protein that is mainly expressed in the liver, and is associated with hepatocyte injury in acute transplant rejection. Reduced levels of FABP1 in mice livers have been shown to be effective against nonalcoholic fatty liver disease (NAFLD). In this study, we investigated the association between plasma FABP1 levels and NAFLD in patients with type 2 diabetes mellitus (T2DM).

**Methods**: We enrolled 267 T2DM patients. Clinical and biochemical parameters were measured. The severity of NAFLD was assessed by ultrasound. FABP1 levels were determined using by enzyme-linked immunosorbent assays.

**Results**: FABP1 levels were higher in patients with overt NAFLD, defined as more than a moderate degree of fatty liver compared to those without NAFLD. Age- and sex-adjusted analysis of FABP1 showed positive associations with body mass index (BMI), waist circumference, homeostasis model assessment estimate of β-cell function, creatinine, and fatty liver index, but showed negative associations with albumin and estimated glomerular filtration rate (eGFR). The odds ratio (OR) for the risk of overt NAFLD with increasing levels of sex-specific FABP1 was significantly increased (OR 2.63 [95% CI 1.30-5.73] vs. 4.94 [2.25-11.48]). The OR in the second and third tertiles of FABP1 remained significant after adjustments for BMI, triglycerides, high-density lipoprotein cholesterol, HbA1C, homeostasis model assessment estimate of insulin resistance, white blood cell count, hepatic enzymes, and eGFR.

**Conclusion**: Our results indicate that FABP1 may play a role in the pathogenesis of NAFLD in patients with T2DM.

## Introduction

Nonalcoholic fatty liver disease (NAFLD) is the most common chronic liver condition worldwide, in part because obesity and insulin resistance lead to the accumulation of triglycerides (TGs) and free fatty acids in the liver. NAFLD ranges from simple steatosis to non-alcoholic steatohepatitis (NASH) characterized by a fatty liver with inflammation and hepatocellular injury 1. Type 2 diabetes mellitus (T2DM) and NAFLD often coexist 2, with a reported prevalence rate of NAFLD of 59.67% in T2DM patients 2. Serial biopsies of patients with diabetes or prediabetes have shown progressive fibrosis 3, and it has also been suggested that the advanced forms of NAFLD such as NASH, advanced fibrosis, cirrhosis, and hepatocellular carcinoma (HCC) occur more commonly in these patients 2. Furthermore, NAFLD is associated with liver-related morbidity and mortality 4, an increased risk of developing adverse cardiovascular diseases 5, and chronic kidney disease (CKD) 6. NAFLD is a metabolic disorder, and its pathogenesis involves complex interactions among hormonal, nutritional and genetic factors 7. In addition, there is a clear association with dysfunctional adipose tissue, obesity, and dysregulated de novo hepatic lipogenesis 8.

Fatty acid-binding proteins (FABPs) are a family of 15-kDa proteins. Nine different FABPs have been identified and named according to the tissues in which they are found 9. FABP1 (also known as liver-type fatty acid-binding protein or LFABP) is expressed mainly in the liver, but small quantities are also found in the kidneys and small intestine 9,10. Previous studies on different types of FABPs have shown that these proteins are associated with tissue damage, including myocardial injury and damage to other organs such as the liver, kidneys, intestine and lungs 11-13.

FABP1 is a 14-kDa protein which is expressed in the hepatocytes and the proximal tubular cells of the kidneys, and participates in fatty acid metabolism in the cytoplasm 14. Furthermore, FABP1 facilitates the transportation, storage, and utilization of fatty acids and their acyl-CoA derivatives and may exert a protective effect against lipotoxicity by facilitating their oxidation or incorporation into TGs and binding otherwise cytotoxic-free fatty acids 15. Some studies on chronic hepatitis C, NASH, and NAFLD have shown that serum FABP1 may be a new diagnostic marker to detect liver injury 16-18. In addition, Petrescu et al. indicated the importance of FABP1 in the fibrate induction of hepatic PPARα LCFA β-oxidative genes, especially in the context of high glucose levels 19. Because NAFLD in patients with T2DM is increasingly recognized to be a public health problem in Taiwan, a study on whether FABP1 is involved in NAFLD is important. Therefore, this study investigated the plasma FABP1 levels in patients with T2DM.

## Methods

### Participants

From January 2017 to December 2018, patients with diabetes who consecutively visited the diabetic or cardiovascular clinics at E-Da Hospital were studied. The diagnosis of T2DM was based on the World Health Organization criteria 20. Patients presenting with symptoms suggestive of type 1 diabetes, defined as diabetic ketoacidosis, acute presentation with heavy ketonuria (3+), or continuous requirement of insulin within 1 year of the diagnosis, were excluded. Patients with a diagnosis of hepatic disease, cardiovascular disease, acute or chronic inflammation, malignancy, and alcohol intake ≥ 30 g/day in men or ≥ 20 g/day in women were also excluded on the basis of interviews and physical examinations. The mean age of the subjects was 67.1±9.7 years, and 68.2% were female. This study was approved by the Human Research Ethics Committee of Kaohsiung E-Da Hospital, I-Shou University (EDAH IRB No. EMRP-106-058). Written informed consent was obtained from each participant before enrolment.

### Data collection

Alcohol intake, smoking habit, medication history, and medical history were assessed using a standardized questionnaire. Body height, weight, waist, and hip circumferences were measured, and the body mass index (BMI) was calculated. The waist circumference was measured at the narrowest point between the lowest rib and the uppermost lateral border of the right iliac crest. The hips were measured at their widest point. Blood pressure was measured in the morning (readings were taken twice, at least 2 minutes apart), on the right upper arm in line with the heart using a mercury column sphygmomanometer with the participant in the sitting position after a minimum rest period of 5 minutes. Patients who had smoked within 1 years of the examination were considered to be current smokers. Those who had stopped smoking for more than 1 year before the examination were considered to be nonsmokers. Most participants were abstainers (88%) or drank minimally (alcohol consumption < 20 g/day; 12% of total). In addition, venous blood was drawn in the morning after an overnight fast. Serum creatinine was analyzed according to the kinetic Jaffé method on a SYNCHRON CX System analyzer (SYNCHRON, Los Angeles, CA) using reagents from Beckman (Beckman Coulter Diagnostic, Los Angeles, CA). Serum TG, total cholesterol, low-density lipoprotein cholesterol (LDL-C), high-density lipoprotein cholesterol (HDL-C), albumin, glucose, and white blood cell (WBC) count were determined using standard commercial methods on a parallel-multichannel analyzer (SYNCHRON, Los Angeles, CA). Hemoglobin A1c (HbA1c) was measured using high performance liquid chromatography. Serum alanine aminotransferase (ALT) was measured following the International Federation of Clinical Chemistry methods.

Estimated glomerular filtration rates (eGFRs) were calculated using the CKD-EPI two-concentration race equation 21, and the status of CKD was confirmed by follow-up eGFR measurements after 3 months. We used the modified National Kidney Foundation classification of CKD 22. In the present study, an eGFR < 60 ml/min per 1.73 m^2^ was defined as CKD, and patients with stage 1 or 2 CKD (eGFR ≥ 60 ml/min per 1.73 m^2^) were classified as not having CKD 23.

### Liver ultrasonography and fatty liver index (FLI) calculation

All abdominal ultrasound examinations were performed by the same specialist. The severity of NAFLD on ultrasound was graded as follows: grade 1 (mild), defined as a slight diffuse increase in liver echogenicity in the hepatic parenchyma with normal visualization of the diaphragm and portal veins; grade 2 (moderate), defined as a moderately diffuse increase in liver echogenicity with a slightly impaired visualization of the diaphragm and portal veins; and grade 3 (severe), defined as a marked increase in liver echogenicity with poor or no visualization of the diaphragm and portal veins. In this study, the subjects with grade 2 or 3 NAFLD were defined as having overt NAFLD.

The FLI was calculated according to a previously published report by Bedogni et al.^24^: FLI = [e^0.953^×log_e_ (TGs) + 0.139×BMI + 0.718×log_e_ (γ-glutamyltransferase (GGT)) + 0.053×waist circumference-15.745)] / [1+ e^0.953^×log_e_ (TGs) + 0.139×BMI + 0.718×log_e_ (GGT) + 0.053×waist circumference-15.745] ×100, with TGs measured in mmol/l, GGT in U/l, and waist circumference in cm.

### Plasma FABP1 and insulin measurements

All blood samples were drawn after overnight fasting, and plasma samples were kept at -80ºC for subsequent assay. The concentrations of plasma FABP1 and insulin were determined using a commercial enzyme-linked immunosorbent assay (ELISA) kits (Cloud-Clone Corp., Katy, USA and R&D Systems, Inc., Minneapolis, USA). The analytical sensitivities were 0.59 ng/mL for FABP1 and 0.881 pmol/L for insulin. ELISA was performed as per the instructions of the manufacturer. According to the manufacturer, the FABP1 ELISA had excellent specificity for the detection of human FABP1, and no significant cross-reactivity or interference with analogues was observed. Samples were measured in duplicate in a single experiment. Homeostasis model assessment estimate of insulin resistance (HOMA-IR) and β-cell function (HOMA-β) values were calculated using equations as previously described 25.

### Statistical analysis

Data normality was assessed using the Kolmogorov-Smirnov test. Continuous, normally distributed variables were presented as mean ± standard deviation, and nonnormally distributed variables as median (interquartile range [IQR] ). Statistical differences in variables were compared using one-way analysis of variance for normally distributed variables, followed by Tukey's pairwise comparison. Before performing the statistical tests, serum or plasma levels of GGT, FABP1, fasting insulin, HOMA-IR, and HOMA-β were logarithmically transformed to achieve a normal distribution. Categorical variables were reported as frequencies and/or percentages, and inter-group comparisons were performed using the chi-squared test. These variables were assessed for independent associations with the presence of overt NAFLD in multiple logistic regression analysis using patients with normal and grade 1 NAFLD as the reference category.

Pearson's correlation coefficients and multiple linear regression analysis were used to examine the correlations and independence between plasma FABP1 and the values of other parameters. In addition, we divided the distribution of plasma FABP1 levels into tertiles in a sex-specific manner. Anthropometric and laboratory data in each tertile were described and tested for trend across plasma FABP1 tertiles by using linear regression analysis. Furthermore, multiple logistic regression analysis was used to assess the odds ratios (ORs) for the presence of overt NAFLD in subjects with higher FABP1 tertiles compared to those with the lowest tertile. Statistical significance was accepted if P < 0.05. All statistical analyses were performed using SAS statistical software, version 8.2 (SAS Institute Inc., Cary, NC).

## Results

### Characteristics of the subjects according to the severity of fatty liver

The duration of diabetes and mean HbA1C levels for all of the subjects overall were 15.1 years and 7.6%, respectively, and 66.7% of the patients had NAFLD. The subjects were divided into three subgroups according to severity of fatty liver disease: normal, grade 1, and grade 2 or 3 (Table 1). The patients with overt NAFLD (grade 2 or 3) had a significantly higher serum FABP1 level than those with grade 1 NAFLD and normal subjects (34.5 ng/mL [IQR 29.6 to 57.1] vs. 29.8 ng/mL [IQR 23.4 to 46.5] vs. 26.6 ng/mL [IQR 20.2 to 38.5], respectively, P = 0.001). In addition, the patients with overt NAFLD had higher rates of hypertension, hyperlipidemia, CKD, angiotensin converting enzyme inhibitor and angiotensin II receptor blocker treatment, and stages 3 and 4 of CKD classes, and higher diastolic blood pressure (DBP), BMI, waist circumference, TGs, creatinine, and FLI than the normal subjects and those with grade 1 NAFLD. Moreover, the patients with overt NAFLD had higher systolic blood pressure (SBP), waist-to-hip ratio, HbA1c, total cholesterol, LDL-cholesterol, aminotransferase (AST), ALT, GGT, WBC count, fasting insulin, and HOMA-IR than the normal subjects. The patients with overt NAFLD also had lower rate of stage 1 of CKD class and lower levels of HDL-cholesterol and eGFR than the normal subjects and those with grade 1 NAFLD. There were no significant differences in age, male gender, currently smoking, oral hypoglycemic agent (OHA) treatment alone, OHA/insulin treatment, stage 2 of CKD class, diabetes duration, fasting glucose, and HOMA-β among the three groups.

### Association between overt NAFLD and clinical laboratory data

In multiple logistic regression analysis after adjustments for age and sex, a high FABP1 level was associated with overt NAFLD (OR 1.02 [95% CI 1.01-1.03]; P = 0.001). In addition, SBP, DBP, BMI, waist circumference, total cholesterol, TGs, HDL-cholesterol, LDL-cholesterol, GGT, AST, ALT, HOMA-IR, HbA1c, eGFR, and WBC count were significantly associated with the presence of overt NAFLD (Table 2).

### Association between plasma FABP1 levels and clinical laboratory data

Pearson's correlation analysis showed that plasma FABP1 levels were positively correlated with age, BMI, waist circumference, fasting insulin, HOMA-β, creatinine, and FLI, and were negatively correlated with albumin and eGFR (Table 3). Furthermore, age- and sex-adjusted analysis of FABP1 showed significant positive correlations with BMI, waist circumference, HOMA-β, creatinine, and FLI, and negative correlations with albumin and eGFR. However, there were no significant correlations between age- and sex-adjusted FABP1 and SBP, DBP, currently smoking, total cholesterol, TGs, HDL-cholesterol, LDL-cholesterol, GGT, AST, ALT, fasting insulin, HOMA-IR, HbA1c, or WBC count.

### Anthropometric and clinical laboratory parameters and overt NAFLD according to the tertile of sex-specific FABP1 levels

To investigate the impact of FABP1 plasma level on anthropometric and clinical laboratory parameters and overt NAFLD, we divided the patients into three groups according to the tertiles of sex-specific FABP1 plasma level. There were significant trends in the associations among FABP1 level and BMI, waist circumference, HOMA-β, albumin, creatinine, eGFR, and FLI (*P* for trend < 0.05) (Table 4). Furthermore, the patients in the second and third tertiles of sex-specific FABP1 had higher ORs for the presence of overt NAFLD compared to those in the lowest tertile (2.63 [1.30-5.73] and 4.94 [2.25-11.48]). The ORs in the second and third tertiles of sex-specific FABP1 remained significant after adjustments for BMI, TGs, HDL-cholesterol, HbA1c, HOMA-IR, WBC count, hepatic enzymes, and eGFR (6.09 [1.11-8.78] and 13.47 [1.79-26.47]) (Table 5).

## Discussion

In the present study, we demonstrated that plasma FABP1 levels were positively correlated with BMI, waist circumference, HOMA-β, creatinine, and FLI, and negatively correlated with albumin and eGFR. In addition, an increased plasma FABP1 concentration was associated with overt NAFLD, even in a fully adjusted model. Furthermore, patients in the highest (third) tertile of FABP1 were 13 times more likely to have overt NAFLD compared to those in the lowest tertile. These findings are in agreement with current evidence regarding the association between NAFLD and FABP1 18.

Liver diseases such as hepatitis, cirrhosis, porphyrias, iron and copper overload, and HCC, are associated with notable changes in cellular lipid metabolic homeostasis, which are usually correlated with changes in cellular FABP levels 26. In the normal liver environment, FABP1 is a key regulator of fatty acid metabolism 27. Serum FABP1 levels are used to monitor fibrosis and hepatocellular damage during liver surgery 28 in both patients with hepatitis C virus (HCV) 16 and NASH patients 17. FABP1 levels are also elevated in human HCC tissues 29, however the elevation observed within tumors does not translate into elevated levels within the blood. Furthermore, serum FABP1 levels are associated with poor survival rates in acute liver failure caused by acetaminophen 30. Despite the strong evidence showing the effect of serum FABP1 concentration on liver diseases, the biological mechanisms by which FABP1 is involved in the pathogenesis of NAFLD are not well understood.

Hepatic oxidative stress plays a key role in the development of NASH/NAFLD development. FABP1 exerts a cytoprotective effect in the liver and kidneys, and it has also been shown to be an effective endogenous antioxidant 15. By binding potentially toxic ligands such as free fatty acids (FFAs) and heme, FABP1 attenuates the detergent effect of FFAs and the generation of reactive oxygen species by heme 15. Moreover, different to other FABP family members, FABP1 exerts a scavenging effect through redox cycling of its methionine and sulfoxide reductase, thereby protecting cells from oxidative stress 31. In contrast, a variety of mouse models and in vitro cell studies have shown that FABP1 regulates fatty acid metabolism associated with peroxisome proliferator-activated receptor alpha (PPARα) in β-oxidation 32, and that it is involved in hepatocellular damage as well as oxidative stress, thus contributing to the progression of liver disease through increased hepatic steatosis and the subsequent activation of hepatic stellate cells 33,34. On the basis of these reports, we think that endogenous FABP1 from hepatocytes may play a significant pathophysiological role in liver disease, and that further studies are required to ascertain the role of FABP1 in patients presenting with NAFLD. In addition, in view of its highly conserved and central role in lipid metabolism and transport of heme and other ligands, further studies are needed to elucidate the role of FABP1 in normal and pathological processes.

Our results showed positive correlations between plasma FABP1 levels and BMI and waist circumference. However, discrepancies in the correlation between FABP1 and obesity have been reported. Shi et al. 35 reported marked increases in FABP1 in healthy obese subjects compared to normal-weight subjects, and that this was strongly correlated with central adiposity. In contrast, two animal studies 36,37 demonstrated that *FABP1^-/-^* mice were protected against obesity when fed a high-fat diet. In addition, a previous review suggested that FABP1 may play an important role in preventing age- or diet-induced obesity 38, and thus that the ''paradoxical'' elevation of serum FABP1 in obese subjects may be compensatory up-regulation to counteract the metabolic stress imposed by obesity. In addition, it is possible that obesity may cause resistance to the action of FABP1 leading to its compensatory up-regulation. Given the cross-sectional design of the current study, no causal inference can be drawn. In addition, Shi et al. also reported that serum FABP1 was positively correlated with insulin resistance in humans 35. In our patients with T2DM, FABP1 was not correlated with HOMA-IR, but it was positively correlated with HOMA-β. Differences in study populations, sex, and FABP1 and HOMA index levels may partly explain these discrepancies.

Our results also showed that the plasma FABP1 levels were positively associated with creatinine and negatively associated with eGFR and albumin in patients with T2DM. Furthermore, higher plasma FABP1 and stages 3 and 4 of CKD classes in grade 2 or 3 of NAFLD was significantly observed compared to normal or grade 1 of NAFLD. FABP1 is expressed in both normal and diseased human kidneys. Two studies of type 1 diabetes 39,40 and three studies of type 2 diabetes 41-43 reported on the relationship between urinary FABP1 concentrations and the severity of diabetic nephropathy. The results showed that in patients with type 1 diabetes, urinary FABP1 concentrations increased with the progression of diabetic nephropathy and were higher in normoalbuminuric patients than in control subjects 39,40. These results indicated that urinary FABP1 accurately reflected the severity of diabetic nephropathy, and that it may be a suitable biomarker for the early detection of diabetic nephropathy. In an animal study, Kamijo-Ikemori et al. 44 found that the expression of FABP1 was markedly increased in diabetic Tg mice at 8 weeks compared with control mice. In addition, hexanoyl-lysine, a urinary marker of oxidative stress, was also significantly lower in the diabetic Tg mice at 8 weeks. Moreover, the levels of macrophage chemotactic and activating factors such as MCP-1 and MCP-3 were significantly suppressed by the expression of renal FABP1, as well as the expressions of TGF-β and α1COL I, which are associated with fibrosis. Furthermore, the expression of FABP1 in the kidneys significantly reduced macrophage infiltration, deposition of type IV collagen, and the progression of tubulointerstitial damage. These results indicate that FABP1 may have a renoprotective function in various renal diseases. Additional studies have also demonstrated that the expression of the FABP1 gene in the kidneys is increased by stress, such as hyperglycemia 44, urinary protein overload 45, renal ischemia 46, and toxins 47, and such stress causes tubulointerstitial damage. FABP1 facilitates fatty-acid metabolism via β-oxidation and causes the excretion of lipid peroxidation products from tubular epithelial cells, thereby inhibiting the release of inflammatory factors and attenuating tubulointerstitial damage to achieve renoprotection 48. Hence, higher plasma FABP1 and stages 3 and 4 of CKD classes in grade 2 or 3 of NAFLD and the positive association between an elevated FABP1 level and creatinine and the negative association with eGFR and albumin in our patients with T2DM may suggest that the higher plasma FABP1 in grade 2 or 3 of NAFLD may be induced by renal dysfunction and elevations in FABP1 level may represent chronic or acute compensatory mechanisms to counteract oxidative stress and inflammation from diabetic nephropathy. These fact had also been observed in many other cytokines reported previously 49. Our study provided evidence that a new cytokine (FABP1), may also be involved in the pathogenic link between NAFLD and CKD. However, the mechanism of action of FABP1 in renal diseases is unclear. Further studies are needed to elucidate the exact role of FABP1 in patients with diabetic nephropathy.

There are several limitations to this study, First, the cross-sectional design limits our ability to infer a causal relationship between increased plasma FABP1 levels and the development of NAFLD. Second, our analyses were based on single measurements of plasma FABP1, which may not reflect the relationship over time. It would be interesting to measure serial changes of plasma FABP1 levels in patients with NAFLD to further clarify the role of FABP1 in the pathogenesis of NAFLD. Third, the severity of NAFLD was assessed using ultrasound in this study, but it was not confirmed pathologically. Although a liver biopsy is the gold standard to assess the pathologic grading of NAFLD, it is difficult to perform liver biopsies to assess NAFLD in clinical practice. A sensitivity of 60-94% and specificity of 84-95% have been reported for ultrasound in the diagnosis of liver-biopsy confirmed fatty liver 50. Fourth, there was no significant association between FABP1 and ALT, AST, or GGT in the present study. A previous study has reported that statistically significant correlations between FABP1 and AST, ALT, and GGT levels 18. However, no association has been reported between serum FABP1 level and AST 51. In addition, the different disease and condition of the study population may have impacted the results.

## Conclusions

In conclusion, we demonstrated that an elevated plasma FABP1 was closely associated with NAFLD in patients with T2DM. Large population-based prospective studies are warranted to confirm whether FABP1 is an independent predictor of NAFLD, and whether it plays a causative role in the pathogenesis of NAFLD.

## Figures and Tables

**Table 1:** Characteristics of the subjects according to the severity of fatty liver

Variables	Normal	Grade 1	Grade 2 or 3	*P*-value
No	89	89	89	
Age (years)	66.8±9.7	68.9±9.7	65.4±9.6	0.050
Male gender, n (%)	24(27.0)	27(30.3)	34(38.2)	0.256
Hypertension, n (%)	0(0.0)	81(91.0)	89(100.0)	<0.0001
Hyperlipidemia, n (%)	21(23.6)	89(100.0)	89(100.0)	<0.0001
Chronic kidney disease, n (%)	12(13.5)	29(32.6)	43(48.3)	<0.0001
Current smoking, n (%)	11(12.4)	12(13.5)	17(19.1)	0.402
Type of treatment, n (%)				
OHA only	68(76.4)	59(66.3)	72(80.9)	0.073
Insulin only	1(1.1)	9(10.1)	5(5.6)	0.034
OHA+ insulin	19(21.4)	21(23.6)	12(13.5)	0.202
ARB and ACEI use, n (%)	30(33.7)	48(53.9)	70(78.7)	<0.0001
Statins use, n (%)	80(89.9)	65(73.0)	54(60.7)	<0.0001
CKD class				
Stage 1 (eGFR ≥90)	24(27.0)	16(18.0)	6(6.7)	0.002
Stage 2 (eGFR 60-89)	54(60.7)	45(50.6)	43(48.3)	0.212
Stage 3 (eGFR 30-59)	9(10.1)	26(29.2)	32(36.0)	0.0002
Stage 4 (eGFR 0-29)	2(2.3)	2(2.3)	8(9.0)	0.043
Diabetes duration (years)	16.3±8.0	14.6±7.6	14.3±6.9	0.163
Systolic blood pressure (mmHg)	128±17	139±17	144±16	<0.0001
Diastolic blood pressure (mmHg)	71±9	76±9	80±10	<0.0001
Body mass index (kg/m^2^)	21.7±2.2	25.8±2.3	30.8±3.8	<0.0001
Waist circumference (cm)	79.4±7.9	89.7±6.7	100.1±7.0	<0.0001
Waist-to-hip ratio	0.88±0.08	0.94±0.07	0.95±0.07	<0.0001
Fasting glucose (mg/dl)	135.0±32.2	144.9±44.0	148.3±43.5	0.074
HbA1c (%)	7.3±1.0	7.6±1.1	8.0±1.7	0.002
Total cholesterol (mg/dl)	170.5±25.7	173.7±27.9	184.9±43.2	0.010
Triglycerides (mg/dl)	70.4±31.1	117.1±48.4	158.7±98.8	<0.0001
HDL-cholesterol (mg/dl)	67.2±17.3	53.0±12.5	51.4±11.2	<0.0001
LDL-cholesterol (mg/dl)	82.3±22.0	89.5±24.3	95.3±36.0	0.009
AST (U/l)	24.6±12.9	27.3±15.2	31.8±19.5	0.012
ALT (U/l)	21.2±10.4	30.8±18.3	36.2±26.1	<0.0001
GGT (U/l)	15.0(13.0-20.0)	25.0(17.5-32.0)	39.0(25.0-70.5)	<0.0001
FABP 1 (ng/mL)	26.6(20.2-38.5)	29.8(23.4-46.5)	34.5(29.6-57.1)	0.001
White blood cell count (10^9^/l)	6251±1492	7286±2091	7468±1844	<0.0001
Fasting insulin (μU/ml)	4.6(4.2-5.2)	5.5(4.8-7.3)	6.4(5.3-9.0)	0.040
HOMA-IR index	1.5(1.3-1.8)	2.0(1.5-2.8)	2.3(1.8-3.6)	0.003
HOMA-β index	25.6(18.3-35.6)	31.9(18.6-43.9)	30.3(19.4-54.0)	0.671
Albumin (g/dl)	4.4±0.2	4.3±0.3	4.3±0.3	0.032
Creatinine (mg/dl)	1.0±0.6	1.0±0.4	1.3±0.9	0.0003
Estimated GFR (ml/min/1.73m^2^)	76.7±19.6	70.5±21.5	60.7±21.8	<0.0001
Fatty liver index	6.2±2.6	35.1±3.3	79.8±8.1	<0.0001

Data are expressed as mean ± SD, number (%), or median (interquartile range). Abbreviations: OHA, oral hypoglycemic agent; ACEI, angiotensin converting enzyme inhibitor; ARB, angiotensin II receptor blocker; HDL, high-density lipoprotein; LDL, low-density lipoprotein; ALT, alanine aminotransferase; AST, aspartate aminotransferase; GGT, γ-glutamyltransferase; FABP, fatty acid-binding protein; HOMA-IR, homeostasis model assessment estimate of insulin resistance; HOMA-β, homeostasis model assessment estimate of β-cell function; GFR, glomerular filtration rate.

**Table 2:** Multiple logistic regression analysis with the presence of overt fatty liver as the dependent variable

Variables	Odds ratios^*^	95% CI	*P*-value
Systolic blood pressure	1.04	1.02-1.06	<0.0001
Diastolic blood pressure	1.06	1.03-1.09	<0.0001
Body mass index	1.94	1.65-2.29	<0.0001
Waist circumference	1.30	1.22-1.39	<0.0001
Total cholesterol	1.01	1.00-1.02	0.012
Triglycerides	1.02	1.01-1.02	<0.0001
HDL-cholesterol	0.96	0.94-0.98	<0.0001
LDL-cholesterol	1.01	1.00-1.02	0.034
GGT	1.05	1.03-1.06	<0.0001
AST	1.03	1.01-1.04	0.004
ALT	1.03	1.01-1.04	0.001
Fasting insulin	1.03	0.99-1.07	0.113
HOMA-IR index	1.15	1.02-1.29	0.026
HbA1c	1.37	1.11-1.68	0.004
FABP 1	1.02	1.01-1.03	0.001
Estimated GFR	0.96	0.95-0.98	<0.0001
White blood cell count	1.00	1.00-1.00	0.011

^*^ Adjusted for age and gender by multiple logistic regression analysis. Abbreviations: CI, confidence Interval; HDL, high-density lipoprotein; LDL, low-density lipoprotein; GGT, γ-glutamyltransferase; AST, aspartate aminotransferase; ALT, alanine aminotransferase; HOMA-IR, homeostasis model assessment estimate of insulin resistance; FABP, fatty acid-binding protein; GFR, glomerular filtration rate.

**Table 3:** Association between plasma fatty acid-binding protein 1 levels and clinical laboratory data

		Model 1			Model 2	
		r	*P*-value		β	*P*-value
Age		0.137	0.026		-	-
Male sex		0.048	0.434		-	-
Systolic blood pressure		0.085	0.166		0.065	0.293
Diastolic blood pressure		0.071	0.245		0.081	0.188
Body mass index		0.218	0.0003		0.238	<0.0001
Waist circumference		0.278	<0.0001		0.271	<0.0001
Currently smoking		0.099	0.105		0.107	0.147
Total cholesterol		-0.007	0.913		0.011	0.857
Triglycerides		0.004	0.951		0.021	0.729
HDL-cholesterol		-0.015	0.811		-0.003	0.961
LDL-cholesterol		-0.002	0.969		0.004	0.947
GGT		0.013	0.833		0.021	0.727
AST		0.012	0.840		0.018	0.773
ALT		-0.049	0.425		-0.047	0.442
Fasting insulin		0.120	0.049		0.113	0.063
HOMA-IR index		0.077	0.212		0.072	0.239
HOMA-β index		0.155	0.011		0.146	0.017
HbA1c		0.023	0.713		0.033	0.596
Albumin		-0.189	0.002		-0.171	0.006
Creatinine		0.375	<0.0001		0.376	<0.0001
Estimated GFR		-0.339	<0.0001		-0.332	<0.0001
Fatty liver index		0.251	<0.0001		0.260	<0.0001
White blood cell count		0.070	0.258		0.071	0.249

Model 1: Pearson correlation coefficient. Model 2: Regression coefficient adjusted for age and sex. Abbreviations: HDL, high-density lipoprotein; LDL, low-density lipoprotein; GGT, γ- glutamyltransferase; AST, aspartate aminotransferase; ALT, alanine aminotransferase; HOMA-IR, homeostasis model assessment estimate of insulin resistance; HOMA-β, homeostasis model assessment estimate of β-cell function; GFR, glomerular filtration rate.

**Table 4:** Characteristics according to the tertile of sex-specific fatty acid-binding protein 1 levels

Parameter	First tertile	Second tertile	Third tertile	*P* for trend
FABP1 (ng/mL)	<24.63 (men), <22.29 (women)	24.63-48.12 (men), 22.29-46.60 (women)	>48.12 (men), >46.60 (women)	
Body mass index (kg/m^2^)	24.7±5.0	26.1±4.4	27.4±4.7	0.001
Waist circumference (cm)	85.6±11.1	89.9±10.4	93.3±11.3	<0.0001
HOMA-β index	30.1(19.2-47.0)	27.2(18.2-38.5)	35.9(20.6-73.7)	0.025
Albumin (g/dl)	4.43±0.26	4.36±0.30	4.26±0.34	0.001
Creatinine (mg/dl)	0.84±0.18	1.02±0.35	1.59±1.45	<0.0001
eGFR (ml/min/1.73m^2^)	84.5±18.5	69.7±19.6	53.9±22.9	<0.0001
Fatty liver index	10.3(5.6-38.0)	35.4(9.2-74.0)	40.8(31.6-82.5)	<0.0001

Data are expressed as mean ± SD, or median (interquartile range). Abbreviations: FABP1, fatty acid-binding protein 1; HOMA-β, homeostasis model assessment estimate of β-cell function; eGFR, estimated glomerular filtration rate; NAFLD, nonalcoholic fatty liver disease.

**Table 5:** Odds ratios for the presence of overt fatty liver according to the tertile of sex-specific fatty acid-binding protein 1 levels

Parameter	First tertile	Second tertile	Third tertile
FABP1 (ng/mL)	<24.63 (men), <22.29 (women)	24.63-48.12 (men), 22.29-46.60 (women)	>48.12 (men), >46.60 (women)
Univariate	1.00	2.63(1.30-5.73)	4.94(2.25-11.48)
Multivariate^*^	1.00	6.09(1.11-8.78)	13.47(1.79-26.47)

Values shown are cut-offs of plasma fatty acid-binding protein 1 levels of all subjects, and odds ratios with 95% confidence intervals. ^*^Adjusted for body mass index, triglycerides, high-density lipoprotein cholesterol, HbA1C, homeostasis model assessment estimate of insulin resistance, white blood cell count, alanine aminotransferase, aspartate aminotransferase, γ-glutamyltransferase, and estimated glomerular filtration rate.
